# Improving the quality of transvaginal ultrasound scan by simulation training for general practice residents

**DOI:** 10.1186/s41077-017-0056-z

**Published:** 2017-11-21

**Authors:** M. Le Lous, N. De Chanaud, A. Bourret, M. V. Senat, C. Colmant, P. Jaury, A. Tesnière, V. Tsatsaris

**Affiliations:** 1 Department of Gynecology Obstetrics and Reproductive Medicine, AP-HP, Cochin Port Royal Hospital, University of Sorbonne Paris Cité, Paris, France; 2 Simulation Department iLumens, Sorbonne Paris Cité University, Paris, France; 3 Department of Obstetrics and Gynecology, University Hospital of Rennes, Rennes, France; 4 General Practice Department, Paris Descartes University, Paris, France; 5 Department of Obstetrics and Gynecology, AP-HP, Bicêtre Hospital, University of Paris-Sud, Orsay, France; 6 Department of Anesthesia, AP-HP, Cochin Port Royal Hospital, University of Sorbonne Paris Cité, Paris, France

**Keywords:** Medical education, Simulation, Transvaginal ultrasound, Gynecology, Ultrasound simulators, General practice

## Abstract

**Background:**

Ultrasonography (US) is an essential tool for the diagnosis of acute gynecological conditions. General practice (GP) residents are involved in the first-line management of gynecologic emergencies. They are not familiar with US equipment. Initial training on simulators was conducted.

The aim of this study was to evaluate the impact of simulation-based training on the quality of the sonographic images achieved by GP residents 2 months after the simulation training versus clinical training alone.

**Methods:**

Young GP residents assigned to emergency gynecology departments were invited to a one-day simulation-based US training session. A prospective controlled trial aiming to assess the impact of such training on TVS (transvaginal ultrasound scan) image quality was conducted. The first group included GP residents who attended the simulation training course. The second group included GP residents who did not attend the course. Written consent to participate was obtained from all participants. Images achieved 2 months after the training were scored using standardized quality criteria and compared in both groups. The stress generated by this examination was also assessed with a simple numeric scale.

**Results:**

A total of 137 residents attended the simulation training, 26 consented to participate in the controlled trial. Sonographic image quality was significantly better in the simulation group for the sagittal view of the uterus (3.6 vs 2.7, *p* = 0.01), for the longitudinal view of the right ovary (2.8 vs 1.4, *p* = 0.027), and for the Morrison space (1.7 vs 0.4, *p* = 0.034), but the difference was not significant for the left ovary (2.9 vs 1.7, *p* = 0.189). The stress generated by TVS after 2 months was not different between the groups (6.0 vs 4.8, *p* = 0.4).

**Conclusion:**

Simulation-based training improved the quality of pelvic US images in GP residents assessed after 2 months of experience in gynecology compared to clinical training alone.

## Background

Ultrasound has become an indispensable tool for the identification of most gynecologic emergencies [1]. However, it is highly operator-dependent. A novice operator can easily miss a diagnosis. In France, during their residency, more than 50% of general practice (GP) residents are assigned to gynecology units for 6 months. They are involved in the first-line management of gynecologic emergencies and have to recognize them [2–4]. Their training field in gynecology lasts one semester, and GP residents have to acquire ultrasound skills rather quickly, especially for a transvaginal ultrasound scan (TVS).

Traditionally, GP residents practice TVS by a combination of observing senior gynecologists and practicing themselves under the supervision of seniors [5]. However, because it is invasive, TVS may create patient discomfort, especially if performed by a novice or, even more, by several operators.

As a response to this problem, new learning methods were developed in the last decade using simulation. These methods represent a solution for teaching TVS without involving any patients, according to the ethical recommendation “never the first time on a patient” [6]. In the context of emergencies, TVS aims to identify the main diagnosis using standardized planes [7]. However, for beginners, the 3D representation, the systematization of US examinations, and image optimization may represent difficulties that could be solved by simulation training [8].

TVS simulators are now available with endovaginal probes. These simulators allow to train to obtain the right planes, know how to zoom, and to optimize the image (gain, depth, and focus) [9].

However, only a few studies have assessed skill transfer from the simulator to real practice [10–12]. To justify the cost of these simulators, it is important to know whether the training has an impact on the management of patients and whether it lasts over time [9]. Tolsgaard et al. did demonstrate a sustained effect 2 months after simulation training on performance of OB/GYN residents, in terms of image optimization, equipment knowledge, systematization of the examination, and image interpretation [13]. TVS images produced by residents are a key element in patient medical files. It is important to assess the impact of simulation on the quality of images produced by GP residents.

The objective of this study was therefore to evaluate the quality of ultrasound images produced by GP residents 2 months after simulation training compared to clinical training alone.

## Methods

This prospective controlled trial study included GP residents in Ile De France who began their semester in the gynecology department in November 2015 or in May 2016. All of the residents were invited to a theoretical lecture on gynecologic ultrasound followed by simulation training at the beginning of their semester in gynecology. Residents who consented to participate in the study were included in the simulation group. Residents who did not show up to this course because of timing conflicts but who volunteered to participate in the study were considered as the control group.

**Fig. 1:**
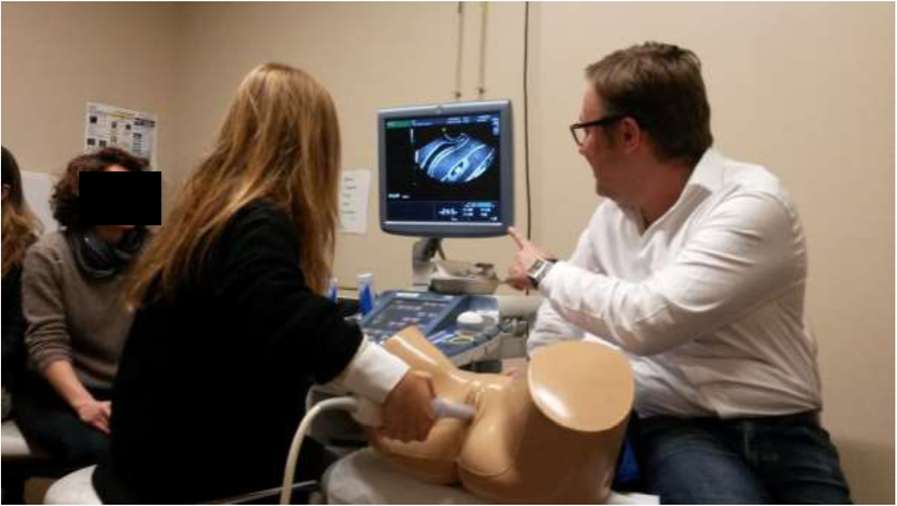
TVS simulators (models)

The theoretical part of the course recalled the physical basis of ultrasound, first-trimester obstetrical pathology, and ultrasound findings in first-trimester pathologies. In the second part, residents trained on pelvic simulators, the Blue Phantom® models (CAE HealthCare, USA, Fig. 1). Real echographs (Voluson E8, General Electric, USA) were used to perform the ultrasound scan on models. Residents were individually trained on the simulator. Instructors who were senior gynecologists taught the residents how to obtain the correct standardized planes in gynecology: the sagittal view of the uterus, right ovary, and left ovary. The Morrison space, or hepatorenal recess defined by the space that separates the liver from the right kidney, was not practiced on models. Different situations were found on the models: ectopic pregnancy, intrauterine pregnancy, and myoma.

In both groups, GP residents practiced TVS in gynecology units in hospitals of Ile De France. The course was conducted 20 days after their starting in the gynecology department. The protocol for all of them was to request help in the case of difficulty in performing the examination or suspicion of pathology.

It was recommended that the residents participating in the study provide one standardized image performed from one patient consulting to the gynecology department for pelvic pain or vaginal bleeding (sagittal view of the uterus, longitudinal view of both ovaries, and Morrison space) within 2 months after the training. The patients were selected by the resident. Their oral and written consent for sharing of images was collected, and images were systematically anonymized.

Images performed in the early pregnancy department were sent after they were anonymized by electronic mail. A visit to their hospital allowed the images of non-respondent residents to be obtained between the second and the third month after the course. The same images were asked to the control group during the same period.

Images gathered after the training were blindly scored by a senior obstetrician according to quality criteria validated by Salomon et al. [7]. These criteria are presented in Table 1. The quality criteria produced by the training group were compared to those of the control group.

**Table 1:** Quality criteria score

Standardized plane	Quality criteria	Points
Sagittal view of the uterus (/4)	Uterus on 2/3 of image surface	1 point
	Fundus of the uterus observable	1 point
	Vacuity line observable	1 point
	Endocervix observable	1 point
Longitudinal right or left ovary planes (/4)	Side specified	1 point
	Ovary on 1/3 of image surface	1 point
	Presence of follicles	1 point
	Iliac vein present	1 point
Morrison space (/3)	Liver and kidney observable	1 point
	Ovoid kidney and not round	1 point
	Both extremities of kidney	1 point

The main outcome was the score obtained on four images performed on real patients 2 months after the simulation training. Eligible patients were women who presented with gynecology emergencies requiring TVS (bleeding or pain in the first trimester of pregnancy).

An auto-evaluation of the residents’ stress generated by TVS was measured using a simple numeric scale from 1 to 10, as a secondary outcome.

Data were stored in an Excel 2013 table and were analyzed with a Mann-Whitney test for quantitative variables with a non-normal distribution and a chi-squared test for qualitative variables using BiostatTGV®.

## Results

**Fig. 2:**
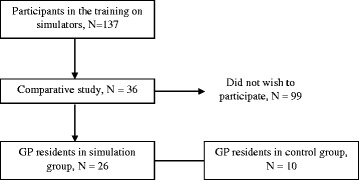
Flow Chart

A total of 137 GP residents participated in the training. Among them, 36 GP residents provided consent to participate in the controlled trial, 26 in the simulation group and 10 in the control group (Fig. 2). Student characteristics were similar in both groups (Table 2).

**Table 2:** Characteristics of students in the simulation and control groups

Characteristics	Simulation group (*N* = 20)	Control group	*p*
	*N* = 26	*N* = 10	
Sex			
Female	19	9	0.27
Male	7	1	
Semester in GP			
1–2	2	0	0.65
3–4	4	2	
5–6	20	8	
Previous experience in ultrasound			
Yes	8	2	0.52
No	18	8	
Interested in gynecology for future practice			
Yes	23	9	0.89
No	3	1	

The quality of images provided 2 months after the simulation training was significantly better in the simulation group for the sagittal view of the uterus (3.6 vs 2.7, *p* = 0.01), for the longitudinal view of the right ovary (2.8 vs 1.4, *p* = 0.027), and for the Morrison space (1.7 vs 0.4, *p* = 0.034). The difference was not significant for the left ovary (2.9 vs 1.7, *p* = 0.189) (Table 3).

**Table 3:** Quality of images after 2 months

Image quality	Simulation group	Control group	*p*
	*N* = 26	*N* = 10	
Sagittal view of the uterus (/4)	3.6	2.7	0.01
Right ovary (/4)	2.8	1.4	0.03
Left Ovary (/4)	2.9	1.7	0.19
Morrison space (/3)	1.7	0.4	0.03

In the simulation group, the global score obtained was satisfactory (quality score > 10) for 70% of the residents versus 20% in the control group (*p* = 0.027) (Table 4). The strict sagittal plane of the uterus was more accessible in both groups, but it was also the plane on which the training had the greatest impact. Ovary examination was problematic for residents who did not attend the training. The percentage of non-satisfactory images (score < 2/4) was 22.5% in the simulation group versus 70% in the control group (*p* = 0.0004). The examination was complete (the presence of four views) for 65% of the trained residents versus 30% of the control group (*p* = 0.15).

**Table 4:** Global score for TVS

Score	Simulation group	Control group	*p*
	*N* = 26	*N* = 10	
Satisfactory (> 10)	17	2	0.01
Non-satisfactory (< 10)	9	8	

The stress experienced by GP residents about performing TVS was not different between the groups at the beginning of the semester (4.8 vs 5.7, *p* = 0.46) versus after 2 months (2.3 vs 3.3, *p* = 0,42) (Table 5).

**Table 5:** Stress of the resident generated by TVS

	Simulation group	Control group	*p*
	*N* = 20	*N* = 10	
Stress at the beginning^a^	4.8	5.7	0.46
Stress after 2 months^a^	4.8	6	0.4

^a^Simple numeric scale

## Discussion

In this study, we found that initial simulation training for TVS permitted to improve the quality of images produced by GP residents in gynecology units after 2 months compared to clinical training alone.

Simulation is one of the options offered by the university for invasive procedures training [14]. The main goal of simulation in obstetrics and gynecology is to train students to procedures involving the genital sphere without mobilizing volunteers [15]. Most women consider TVS to be at least uncomfortable [16]. Task trainers for teaching vaginal digital examinations already exist. TVS simulators recently appeared and represent a revolution for the residency curriculum [17].

A metanalysis by Cook et al. compared the effectiveness of technology-enhanced simulation versus traditional training. Simulation training was effective for student satisfaction, knowledge, and skills [18]. Skill improvement on simulators was therefore expected. However, transfer of skills to clinical practice and patient effects after simulation training are not well known [19]. The Kirkpatrick pyramid can be used to rank medical education studies depending on the main outcome in four levels: 1—student satisfaction; 2—progress in theoretical and practical knowledge outside the clinic; 3—impact on clinical skills; and 4—impact or benefit for the patient [20]. As our primary outcome is image quality when performed on real patients, our study can be ranked as level 3.

To justify the costs that are invested in this type of training [9, 18, 21], it is necessary to know whether simulation training has a sustained impact on trainees’ skills [22]. Although an immediate effect of simulation is well known, only a few studies have assessed the long-term retention of the skills [23]. In a randomized prospective study, Tolsgaard et al. assessed the sustained effect of TVS simulation training using the Objective Structured Assessment of Ultrasound Skills (OSAUS). Ultrasound competence can be assessed using this valid score. The pass/fail scores may be used to help determine whether trainees are qualified for independent practice [24]. In this study, Tolsgaard et al. Demonstrated that simulation allowed significantly better image optimization, better approach systematization, better image interpretation, and a better decision-taking process with a sustained effect at 2 months. Participants reached the expert level on the simulator in a mean time of 3 h 16 min [13].

Other researchers have reported positive impacts of ultrasound simulation in residency curricula in other areas [25]. In traumatology, for instance, the ultrasound skills of GP residents trained by simulation were similar to those who trained on real patients [26].

In our study, we focused on GP residents. Their training field in gynecology is short, but the flow of patients is huge. They have to become autonomous very quickly. They are not familiar with TVS. The number of US scans necessary to acquire competency in first-line TVS is not known [11, 27]. It is definitely not necessary to teach GP residents expert TVS. However, some diagnosis in gynecology (pregnancy localization, hemoperitoneum) are easily identifiable thanks to a few standardized planes [3]. The four planes that are required are the sagittal view of the uterus, longitudinal views of both ovaries, and the Morrison space [28].

Another advantage of simulation training is that students face many conditions in a very short time. The models showed various situations: intrauterine pregnancy with the possibility of determining gestational age, myoma, ectopic pregnancy, and hemoperitoneum.

Our study had methodological weaknesses, mostly because the number of participants was small. There was no sample size calculation. Another bias was the absence of randomization of the groups. Residents who volunteered for the study were recruited after they did or did not show up to the courses. Our selection was therefore prone to bias. Plus, selection of patients to collect images from for assessment was not standardized. Despite those bias, it was noteworthy that the residents in the simulation group had a significantly better systematic approach and more respect for the standardized quality criteria.

Patient benefits of ultrasound simulation in the area of gynecology, in terms of morbidity or mortality, have not yet been demonstrated. Further research in this area is needed. However, one can assume that better systematization of TVS may avoid misdiagnosis (cysts complications, ectopic pregnancies).

Other benefits for patients were demonstrated in a previous study by Tolsgaard et al. During TVS, patients felt less discomfort and more confidence during TVS when the operator was trained by simulation [29].

Finally, in the area of education, Chalouhi et al. suggested that the use of ultrasound simulators was possible for the National Examination. Indeed, images obtained by residents on simulators were correlated to those obtained on real patients [30]. This implies that volunteers for National examination are no longer necessary.

Simulation has its place in the initial training of GP residents in gynecology. Studies have shown benefits in gynecology and in obstetrics for fetal defect screening. The benefits in terms of public health and impact on the detection of pathologies are, however, difficult to evaluate.

## Conclusion

Simulation-based training improved the quality of pelvic US images in GP residents assessed after 2 months of experience in gynecology compared to clinical training alone
